# Intelligent dynamic cybersecurity risk management framework with explainability and interpretability of AI models for enhancing security and resilience of digital infrastructure

**DOI:** 10.1007/s40860-025-00253-3

**Published:** 2025-07-09

**Authors:** Shareeful Islam, Nihala Basheer, Spyridon Papastergiou, Mario Ciampi, Stefano Silvestri

**Affiliations:** 1 School of Computing and Information Science, Anglia Ruskin University, East Road, Cambridge, UK; 2 Institute for High Performance Computing and Networking, National Research Council of Italy (ICAR-CNR), Via Pietro Castellino 111, 80131 Naples, Italy; 3 Research and Innovation, Maggioli S.p.A., Santarcangelo di Romagna, Italy; 4 Department of Informatics, University of Piraeus, Pireas, Greece

**Keywords:** Cybersecurity, Risk assessment, Artificial intelligence, Explainability, Interpretability

## Abstract

The sophistication of cyberattacks has significantly increased, making it almost certain that organizations can be victims of cyberattacks at any time. Managing cybersecurity risk is critical for any organization so that informed decisions can be made to tackle risks before they materialize. Cybersecurity risk management is context-specific and heavily relies on the specific organization’s context. However, performing effective risk management is always challenging due to the constant changes in organizational infrastructure and security posture, including the adoption of new applications and the reconfiguration or updating of existing assets and their dependencies, as well as the potential exploitation of vulnerabilities. Despite the wider adoption of AI enabled cybersecurity risk management, there is a lack of focus on the integration of these systems along with the dynamic elements of the risk management. In this context, this research proposes a novel dynamic cyber security risk management (d-CSRM) framework to tackle this challenge by integrating dynamic parameters such as vulnerability exploitation and assets dependencies for assessing and managing the risk. The framework consists of a systemic process and makes use of a hybrid AI-enabled model that combines both linear regression and deep learning, to prioritize the vulnerabilities. Additionally, d-CSRM integrates the explainability and interpretability characteristics of the AI model for explaining model decision making and the inner working parameters. This allows the extraction of the key features that are linked with the risk and informed decision making to tackle the risks. An experiment was performed to prioritize the vulnerabilities from the widely used CVEjoin dataset using the proposed hybrid model to quantify the dynamic risk with explainability. The results show that the hybrid model effectively identifies and prioritizes the most critical vulnerabilities using the selected key features such as exploit type, exploit platform and impact that can further enhance the dynamic risk assessment.

## Introduction

Organizations are now heavily relying on digital technologies, which provide significant benefits, including more efficient and coordinated service delivery, higher degrees of flexibility, and scalability of the overall infrastructure. Despite these benefits, the infrastructure within an organizational context is now more complex, with growing interconnectedness among different sub-systems that create attack surfaces for potential risks. Hence, a cyber-attack from any part of the system can propagate to other parts of the infrastructure, posing potential disruptions [1]. Organizational cyberspace is now continuously evolving due to the rapidly changing infrastructure, including applications, technologies, and devices [2, 3]. Moreover, the security context is also evolving, with the threat landscape continuously changing, new attack surfaces emerging, and a higher likelihood of published vulnerabilities being exploited.

In this context, there is a need to update the cybersecurity risk status based on temporal parameters relating to organizational cyberspace, such as vulnerability exploitation, asset dependencies, attack paths and many more [4, 5]. In particular, a large number of vulnerabilities are reported every day, but only 2% of the published ones are observed to be exploited [6, 7]. Moreover, the existing vulnerability scoring system lacks the focus to evaluate the probability of exploitation. Accurate risk estimation requires considering the vulnerabilities that are likely to be exploited in a specific organizational context [8]. Despite existing works and industry practices that provide comprehensive risk management frameworks, there is a lack of focus on identifying and assessing risks that arise from temporal parameters [2, 3, 9–12].

Furthermore, AI-enabled cybersecurity risk management is now considered within risk management due to the large volume of data associated with different components, such as log and vulnerability data. This facilitates the model to undertake proactive course of action for the risk mitigation. However, there is a lack of focus on explaining and interpretating the model decision making and outcome [13]. This can hinder trust and accountability, complicating efforts to justify and validate the actions recommended or taken by the AI systems [14]. To address this issue, explainability and interpretability can be incorporated to provide transparency of the model, allowing cybersecurity teams to understand and trust the AI’s recommendations, thereby enhancing the effectiveness and accountability of AI-driven risk management [13]. This certainly helps to develop a fortified defense mechanism against advanced threats and along with it supports business continuity by protecting key assets from new emerging cyber risks. Therefore, it is necessary to incorporate explainability and interpretability for the AI-enabled cybersecurity risk management to increase the trust in the model results and to improve the effectiveness of risk mitigation methods.

The proposed work advances existing risk management practices by adopting temporal parameters to understand the organizational and security context, and hybrid AI-enabled models to assess and manage risk. The work incorporates the explainability and interpretability to enhance the understanding of the model decision making, outcomes and inner working. Hence, this work presents a dynamic cyber security risk management (d-CSRM) framework to understand the overall evolving organizational and security context for effective risk management practices. The d-CSRM presents three novel contributions.

- Firstly, the framework includes a systematic process that considers the temporal parameters of the organizational system and security context, i.e., vulnerability exploitation, asset dependencies, attack complexity, security objective impact, and common attack point to assess and manage risk. Both individual and cascading risks are considered based on the independent and dependent assets within the overall infrastructure.
- Secondly, it uses a hybrid model that combines both linear regression and deep learning for vulnerability prioritization. The benefits of using ensemble technique for the hybrid model is that it can measure feature importance that are linked with the temporal parameters of the risk assessment [15]. The selected features are then combined with deep learning model to predict the vulnerability exploitability score and prioritize the vulnerabilities for the risk estimation.
- Thirdly, d-CSRM incorporates the explainability and interpretability characteristics of the hybrid model. This allows for systematically explaining and understanding the model decision making process and inner working of the model. The SHAP (SHapley Additive exPlanations) along with LIME (Local Interpretable Model-Agnostic Explanations) framework are used to explain the model predictions while expressing the impact of individual features and interactions between them. The incorporation of explainability and interpretability in the hybrid model can enrich the efficiency and performance of the model by enabling us to modify the model with the required changes to its hyperparameters.
- Finally, an experiment is performed to determine the applicability of the hybrid model and overall d-CSRM. The experiment considers the CVEjoin dataset, which includes distinct features for presenting vulnerability from both NVD and various other security knowledge base [16]. The result from the experiment shows that that the proposed hybrid model performs with a relatively low mean squared error (MSE) value and able to prioritize the vulnerabilities.

## Related works

### Cyber security risk management and standards

Cybersecurity risk management is essential for organizations that are experiencing an increase of cyber threats, where the implementation of standards is known to amplify the probability of cyber assurance [17]. There are several international standards that provide best practice guidelines for the organizations to assess and manage risk [18]. Since these standards offer comprehensive and harmonized information on risk identification, analysis, evaluation, treatment, and control, it ensures enhanced cybersecurity maturity in organizations’ defense against cyber-attacks [19]. ISO 31000 provides foundation for risk management practices, independent of any sector with the consideration of organization’s internal and external environment for risk management [20]. ISO 27005 is the key reference standard identifying and assessing information security risk management process [21]. It defines a management process that analyses the risks and categorizes them according to a priority level. The National Institute of Standards and Technology’s SP 800-39 (NIST 800-39) provides framework for multitiered based information security risk management from three distinct tiers: organizational, mission/ business, and information system context [22]. The risk framing considers the risk management context with the tiers before assessing and managing the risks.

An integrated cybersecurity risk management framework is proposed by Kure et al. that make use of the common security knowledge to understand the threats and vulnerabilities related with the assets and machine learning models for the risk type predictions [9]. The framework includes a conceptual language and systemic process to define integrated risk management activities along with tool support to automate the whole process. A smart grid case study is used to evaluate the framework, where several risk categories are identified and controlled. Cherdantseva et al. [10] review the existing risk management practice tailor for the SCADA systems based on sector, risk management principles, evidence, and others. The review outcome emphasizes on the holistic aspect of the risk management. Kanakogi et al. [11] proposed an automate tracing method from CAPEC-IDs to CVE-IDs through CWE using TF-IDF and Doc2Vec. TF-IDF measures the importance of a word in a document relative to its frequency across a collection of documents, while Doc2Vec generates vector representations for entire documents to capture their semantic meaning. Additionally, they experimentally confirm that TF-IDF is more accurate than Doc2Vec. The method would be useful to understand the common security knowledge for risk management, but it is not applicable to all patterns.

Risk assessment methods have quickly become obsolete in dynamic environments, which lead another dimension of work towards dynamic risk management. Naumov and Kabanov propose a dynamic risk assessment framework to better capture cyber-related risks [3]. It offers updating policies and standards to address modern cyber risks effectively. A review of current state of the dynamic risk management methods is performed by Cheimonidis and Rantos [2]. The outcome of review shows that most of the approaches adopt AI/ML mode for the risk management with focus on industrial control system, supply chain and ICT domain. Additionally, the methods are classified accordingly three distinct mature level and a number of limitations observed including lack of consideration of CTI related information and historic data for risk assessment. Granadillo et al. propose a Dynamic Risk Management Response System (DRMRS), featuring proactive and reactive management software designed to evaluate threat scenarios automatically and anticipate potential attacks [23]. The approach dynamically assesses the scenarios based on likelihood of threats and related impact, response costs, and any negative side effects.

### AI-enabled cyber security risk management

The adoption of AI in cyber security risk management is widely considered as one of the vital components of cybersecurity practice. This is due to the fact that AI can improve threat identification and response, as well as risk mitigation [24, 25]. Technology like machine learning and natural language processing can strengthen identification and countermeasures various types of threats like malware, phishing attacks, and insider threats [26, 27]. Pandey and Katsikas [28] propose AI-based, DLT integrated smart contract platform for managing risk models, risk mitigation, and constructing RT instruments, and for prompt settlement. The integration of AI and DLT technologies is planned to increase the security and efficiency of managing cyber risks. Ligo et al. [29] introduces an Autonomous Intelligent Cyber Agents (AICA) which aims to detect attack in real time and act across enormous scale at the same time. Jyothi et al. [30] proposes high-security safe data access management system AI to counter cyber threats in an efficient manner. It underlines the need to have security measures and good handling of data within the period of the delivery of services online and use of electronic devices. The authors of [27] propose a NLP method based on large language models and machine learning (XG-Boost) to obtain threat and vulnerability assessment for the healthcare ICT assets, exploiting constantly updated information crawled from the web and cybersecurity Knowledge Bases. Another NLP-based approach for cyber threat assessment and management is described in [31, 32], allowing to assess the threats related to several real-world scenarios by leveraging natural language documents crawled from cybersecurity news websites, demonstrating its effectiveness and usability.

In summary of existing contributions and risk management standards, there is lack of consideration of dynamic parameters from organizational and security context for the risk assessment and management activities. This could impact on inaccurate risk identification and estimation. Additionally, AI enabled cybersecurity risk management is widely considered by the research community and industry community but limited consideration of explaining the decision making from the models. Without understanding the decision making, model could provide inaccurate results and users cannot make informed decision making for managing the risk. In this context, the proposed d-CSRM addresses these limitations by incorporating explainability and interpretability characteristics for the AI models. This allows the extraction of the important features and inner working parameters of the model within the risk management context.

## Explainability and interpretability for AI-enabled models

Despite the widespread use of AI, it poses significant challenges especially with respect to accountability and comprehensibility. Moreover, there are concerns regarding the ethical and legal considerations of the models about its decision-making. The EU AI Act focuses on accountability for AI systems, their use, and the incorporation of ethical practices [33]. This is to ensure that AI models will always be transparent, and the steps they undertake in their decision-making processes are also explainable by a human. In this respect, explainability and interpretability are the possible solutions to rectify the opaque nature of AI models, as they make AI more comprehensible. Explainability and interpretability are one of the crucial components of any AI model as they enable users to understand all the decisions made by the model [34]. They aid in identifying and correcting biases, ensuring the ethical use of AI, and meeting regulatory requirements that mandate clear explanations for automated decisions. Additionally, it aids in increasing the trust in the recommendations or predictions provided by the model. In other words, explainability focuses on providing a comprehensible explanation for the decisions made by the model, while interpretability focuses on explaining the internal workings of the model.

### Interpretability for AI models

The term interpretability in the context of AI relies on the understanding of, and clearly explaining the logic of a model’s decisions. It makes the mapping of the input features to the result of an AI model as transparent as possible so that the users can understand the path taken by an AI model. Such a practice is necessary for establishing credibility and trust, especially for high stake applications such as risk management, healthcare, and finance. Interpretability helps in improving models by ensuring ethical AI practices by identifying and mitigating biases, and complying with regulatory requirements that demand explanations for the decisions made [32]. The different techniques that can be used to ensure interpretability are by using simple, naturally interpretable models like linear regression and decision trees, and utilizing model-agnostic methods. In this way, interpretability is improving AI models by contributing to the creation of less error-prone, more responsible AI solutions.

**Fig. 1:**
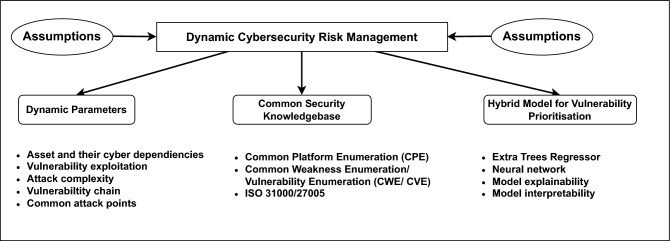
Key components of the proposed dynamic risk management method

### Explainability for AI models

Explainability, when used in relation to AI models, means the ability to explain how an AI model arrives at its decision or prediction. This involves presenting relevant information in plain language and making it easy for the user to understand how the model works, the data that is being fed into it, and what is being produced by the model. This is critical to ensure trust in the recommendations of the AI [35]. Another feature of explainability is transparency since the model’s output explains how the AI model arrives at a certain decision [36]. This understanding is crucial for cybersecurity analysts to ensure the actions taken by AI are accurate and to learn to trust the AI system. AI models come with great responsibilities, and organizations must be able to justify why their AI model performed a certain action. Explainability achieves this by providing insights into what the model does and the decisions it made. It also means that organizations that adopt explainability maintain compliance with regulations to avert legal and reputational pitfalls. In the context of cybersecurity, explainability could vary depending on the type of dataset and model used:

- *In case of source code type datasets*: Explainability in the context of source code datasets entails the process of pinpointing the weaknesses within the code. AI models scan through the source code and give specific lines or segments of the code that contain some risks. They have the ability to recognize patterns or behaviors that could indicate insecure coding practices such as functions that are deprecated or areas which are vulnerable to buffer overflow.
- *In case of Threat intelligence type dataset*: For threat intelligence datasets, explainability is related to understanding how AI models determine newly emergent threats. These datasets have dynamic data with regards to threats and how they intend to attack. This information is then combined with the static vulnerability data, and explainability methods are used to demonstrate how various threat intelligence inputs are used to make predictions.
- *In case of API type dataset*: In the context of API-related datasets, explainability demonstrates how the AI model links API requests and responses to reveal undesired actions, e.g., frequent, or unexpected calls. The model’s decision making is further made transparent by pointing out which API calls are considered malicious.
- *In case of log dataset*: Such primary sources as log datasets that include comprehensive information about the activities of systems and networks are important for the detection of security threats. In this context, explainability means how the AI model is used to identify certain activities that are considered anomalous or to indicate that some vulnerability is being exploited.

## Dynamic cybersecurity risk management (d-CSRM)

The proposed d-CSRM contributes beyond the existing risk management practice by considering dynamic parameters based on organizational and security context for both individual and cascading risk assessment and management. This section provides an overview of d-CSRM including the key components, assumptions, and process.

### d-CSRM key components

d-CSRM is formulated by four key components which provide the foundation for the risk management activities as presented in Fig. 1: *assumptions*, *dynamic parameters*, *common security knowledge*, and *hybrid model for vulnerability prioritization*.

#### Assumptions

We have considered a list of assumptions that are necessary to consider developing the proposed method:

- The organizational underlying digital infrastructure consists of the interconnected ICT infrastructure including systems, applications, devices to support the overall business processes. Cybersecurity risks need to be considered from holistic aspects and associated with the entire ICT infrastructure and other non-technical components.
- The proposed methodology considers dynamic parameters from the organizational and security context such as assets and their dependencies, vulnerability exploitations, common attack point, etc for the risk assessment and management.
- The d-CSRM performs the risk assessment and management activities for both individual and cascading risk. The cascading risks are considered only for dependent assets and propagation of vulnerabilities within the dependent assets.
- The proposed methodology should follow systematic sequential phases and steps within a specific phase that makes the overall process usable and easy-to-learn.
- The d-CSRM should encapsulate complete and accurate domain-specific concepts, related to risk management, cyber security, and sector specific concepts. The relevant concepts support reasoning and analysis of the identified risks for an effective risk management practice.

#### Dynamic parameters

As stated before, d-CSRM considers several parameters which are evolving over time and impact on the risk level.

- *Assets and their dependencies*: Assets are the key entities with varying criticality that have value for the organization. It can be hardware, software or any other ICT infrastructure which are essential to delivery services and support entire organizational business process. Assets are dependent upon each other to support specific process or services within the organization. These interactions have evolved within the cyberspace of the organization which certainly impact the risk estimation. d-CSRM considers both service and cyber level dependencies so that interaction among the assets can be identified and considered for the risk identification.
- *Vulnerability exploitation*: Vulnerability is known as a weakness or flaw in a product that can be exploited by a threat actor for a successful cyber-attack. Common vulnerabilities and exposures (CVE) is one of the key areas of common security knowledge, which publishes product-specific single or multiple vulnerabilities. CVE includes Common Vulnerability Scoring System (CVSS) 4.0, which is widely used to determine the severity of the vulnerability. However, CVSS score is static and does not focus on the likelihood of the vulnerability being exploited, which makes it challenging to determine which of the published vulnerabilities are relevant to the specific context. In this regard, Exploit Prediction Scoring System (EPSS) is a data-driven system that estimates the probability of the vulnerability being exploited based on observed evidence of exploit code or PoC in various data sources such as Exploit-DB, Rapid7, GitHub etc. d-CSRM considers vulnerability exploitation as a dynamic parameter because exploitation is dynamic in nature.
- *Attack complexity*: The exploitation of vulnerability depends on the complexity of the attack. In the case of high complexity, it is challenging to exploit the vulnerability and vice versa.
- *Vulnerability chain*: It is a type of cyberattack that group multiple exploits based on the dependent assets to propagate vulnerabilities aiming to compromise a target asset. Such attack requires advanced skills and effort by the threat actors and could have a systemic catastrophic consequence. The vulnerability chain relies on attack path discovered, including entry point, intermediate and target asset along with their dependencies and related vulnerabilities that can propagate through the assets. Therefore, these chains have evolved due to the variation of dependencies of the selected assets and different level of vulnerability exploitation of the related assets.

#### Common security knowledge and standards

As stated before, we have adopted a number of common security knowledge bases and standards to support the threat and risk assessment, and management process. This approach enables us to feed our asset-threat-vulnerability model with legitimate data sources like CPE, CWE, and CVE and relevant standards such as ISO 31000 and 27005. An overview of the knowledge base is given below:

- Common platform enumeration (CPE) [37] is a standardized format to enumerate the software, platform and other assets. CPE makes it easier to map the various components residing in systems and various scanners used for enumeration to utilize the CPE catalogue for their output.
- Common weakness enumeration (CWE) [38] catalogue is a community-developed source of software and hardware weakness types. Weaknesses can be flaws, faults, bugs, and other errors in software and hardware design, architecture, code, or implementation that has the potential to make systems, networks, and hardware vulnerable to attacks. The CWE catalogue aims to support both the development and the security practitioner communities.
- Common vulnerabilities and exposures (CVE) [6] provides accepted and published list of vulnerability. Each CVE entry contains a unique ID, description, reference, severity scores, and impact ratings according to the Common Vulnerability Scoring System (CVSS) [39]. CVSS provides numerical score, reflecting the severity of the individual vulnerability which can be used to prioritize the vulnerability relevant to a context.
- Risk Management Standard-ISO 31000:2018 [21] provides guidelines for risk management independent of any sector, whereas ISO/IEC 27005:2022 [20] focuses on information security risk management activities. Both standards follow a process-based approach with systematic sequential activities for risk assessment and management.

**Fig. 2:**
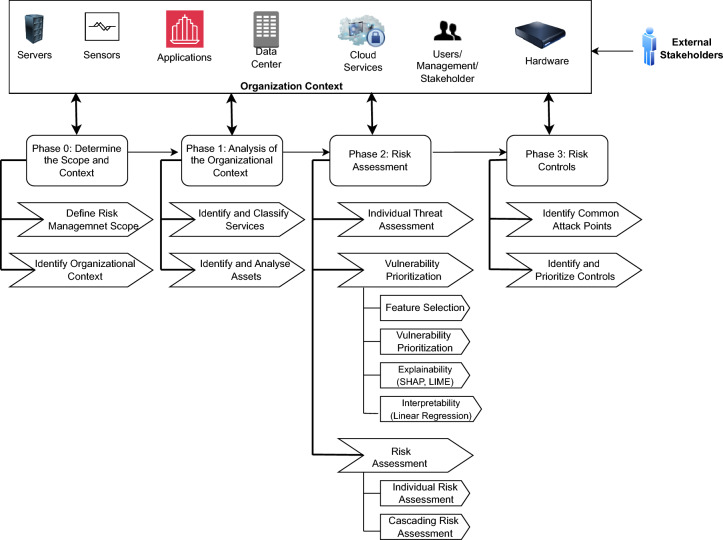
Phases and steps of the d-CSRM

#### Explainability and interpretability of AI model for d-CSRM

The proposed hybrid model integrates the strength of both linear regression and the deep learning model to predict EPSS score and then prioritize the vulnerability. Linear regression is used to analyze the linear associations between the features, thus, being quite interpretable, while deep learning models estimate intricate, non-linear dependencies. This combined with each other improves the predictability of the results and the possibility of their further analysis that allows creating a reliable system of vulnerability management. AI models, especially when it comes to prioritizing vulnerabilities, are irreplaceable due to their ability to manage and analyze the vulnerability exploitation with ease. Such models, particularly deep learning models with multiple layers and non-linear transformations, are capable to identify complicated patterns of the features and their impact in vulnerability exploitation. The use of AI models becomes essential because the large-scale volume of data that needs to be processed are beyond the human capabilities. However, one of the important aspects of the AI models is its ability to explain and interpret the decision of the models and its outcome.

To enhance the explainability, SHAP and LIME framework were utilized for this research. SHAP values provide a standardized value for the features that help explain how each feature affects the model’s predictions in different instances [40]. LIME provides the local approximation of the model to explain the prediction of individual instances to the users [41]. On the other hand, linear regression is applied for interpretability due to its ability to understand how the input features are related to the output. This enables the hybrid model to not only improve the accuracy and credibility of the predictions but also makes the decisions made by AI transparent and comprehensible. It also improves the trust in the model results and makes the vulnerability management easier.

### d-CSRM process

The process comprises of four sequential phases of activities and steps within the phase that an organization can follow for assessing and managing risks. It provides a roadmap for organization to achieve overall security and resilience. The initial two phases focus on defining risk management and comprehensive understanding of the organizational context. While the final two phases focus on calculating the individual and cascading risk and determining the necessary controls to tackle the risks. We follow the guidelines of the existing risk management standards such as ISO-31000 and 27005 to define the process. These four phases, including the corresponding steps, are summarized in Fig. 2.

#### Phase 0: determine the scope and context

This is the first phase defines the overall risk management scope based on the specific organizational context which requires active involvement of key stakeholders, such as operation manager, IT and security manager, and business analysts, to define the overall risk management scope. This phase includes two steps, i.e., define risk management scope and identify organizational context.

**Step 0.1: Define risk management scope**

The step defines the scope of the risk management activities in terms of objectives, risk types and acceptance criteria. Generally, there are different types of risks associated with the entire organizational ecosystem and the scope identifies the relevant risk types, which are taken into consideration for the methodology. A typical risk management scope can be derived from the security and resilience, privacy, business continuity, and operational perspectives. It is also necessary to define the risk criteria for specific risk types in terms of risk level quantification and acceptance for a specific risk level within specific organizational settings. The criteria depend on the nature and type of risks affecting the overall business continuity. The scope enables us to choose the relevant resources for the risk management activities. The outcome of the step is the definition of the cyber security risk management scope.

**Step 0.2: Identify organizational context**

The organizational context defines the boundary of the risk management scope and activities. In general, risk management takes place in the context of the objectives and activities of the organization (ISO 3100 [21]). Therefore, this step identifies both internal and external factors or rules within which the organization is operating. The context plays an important role for the risk management activities and determines the appropriate risks, which ensures that suitable control actions can be taken into consideration. Hence, all risks should be defined within the context of the organization:

- The internal context relates to the factors within the organization that influence the overall internal operational environment, including resources, services, internal stakeholders, organization structure and culture, agents, data flow, data repositories, guidelines, code of ethics, and many more.
- The external context relates to the factors from outside of the organization that influence the organization, including relevant laws and legislation, government policies, supply chain institutions, suppliers, political and economic situations.

The outcome of this phase is a risk management plan that includes the overall scope and context with the following set of attributes:

- *Scope*: The aim and objective of the risk management activities mainly focus on security, privacy, resilience, and operation of the service delivery of the organization. It also includes the type of risks that need to be taken into consideration for the methodology.
- *Internal context*: Factors relating to the internal environment of the organization.
- *External context*: Factors relating to the external environment of the organization.
- *Risk criteria*: The criteria or rule set used to determine the risk level and its acceptance. For instance, risks having low impact could be potential for acceptance.
- *Roles and responsibility*: The roles involve performing the risk management activities and communicate the risk-related information to the relevant parties.

#### Phase 1: analysis of the organizational context

Once the risk management scope and context are defined, the next phase is to analyze the organization’s context. The aim is to identify the services and assets along with their dependencies so that risk related to the services and assets can be assessed and management. This phase consists of two steps which focus on identifying services and assets related with the services.

**Table 1:** Asset cyber dependencies

Source asset	Destination asset	Asset cyberdependency	
		Dependency type	Access vector
$a_{r,m}$	$a_{l,m}$	Installing	Local (L)
$a_{p,t}$	$a_{q,t}$	Hosting	Local (L)
$a_{q,t}$	$a_{r,m}$	Exchange data/information	Network (N)

**Step 1.1: Identify organizational context**

The first step focuses on the identification of the available services that are necessary to run the business process. Each business process is part of a specific system and may depend on external entities for its operation. These dependencies need to be taken into consideration for asset and risk identification. The identified services are classified into four different types to determine their criticality, which are given below:

- *Business supporting*: Such services have been developed over time to deal with small, recurring issues or functions. In the case of an incident, they will not be missed in the short term and certainly not while business operations are being recovered. These services will need to be recovered over the long term. For instance, general information about a hospital website. The recovery time requirement for these types of services is often measured in weeks or perhaps even months.
- *Business operation*: These services are more prioritized than supporting services and will not stop the organization from functioning in the short term. However, the unavailability of these services could have a long-term potential impact and may cause some disruption to the business. For instance, remote telephone consultations for patients or resource allocation for a support function within a healthcare system. It is necessary to take a moderate amount of time and effort (as compared to mission-critical services) to restore these to a fully functioning state. The recovery time requirement for important business processes is often measured in days or weeks.
- *Business critical means*: A business-critical service is one that an organization relies on to carry out the normal operations that keep it running successfully. When a business-critical service fails or is interrupted, organizations can face financial losses, customer dissatisfaction, and reductions in productivity. From a healthcare perspective, these services may include medical reports, prescription requests, and deliveries. These are vital services for the organization’s operation. The recovery time requirement for such business processes is often measured in hours or days.
- *Mission critical means*: Mission-critical business services are those that have the greatest impact on the organization’s operations and potential for recovery. The difference between mission-critical and business-critical lies in the major adverse impact and the very real possibilities of loss of life, serious injury, and/or financial loss. When a mission-critical system experiences an outage, the results could be catastrophic, whereas business-critical impacts are mainly economic, such as lost customers or breached contracts.

**Step 1.2: Identify and analyze assets**

Once the services are identified, the next step is to identify and analyze the assets that are essential for these services. This step utilizes the NIST CPE catalogue, where known components are cataloged with a specific CPE URI, which can be considered as asset-specific details [42]. Each identified asset is categorized based on three distinct characteristics:

- *Type*: Software, hardware, Operating System (OS), information, etc.
- *Sensitivity*: Restricted, unrestricted.
- *Importance*: Essential, required, deferrable.

An asset may be involved with single or multiple services; therefore, it is necessary to categorize the assets based on their dependencies. d-CSRM considers four distinct types of service-level dependencies to categorize the assets. They are:

- *Independent*: Assets may have a distinct operation and exhibit no dependency on other assets. If the asset fails, no cascading events occur
- *Incoming*: An asset may have an incoming dependency on another asset that utilizes its data or functionality. If such an asset fails, the operation of all related assets that utilize its data or functionality could be impacted, potentially causing disruption.
- *Outgoing*: Assets may have an outgoing dependency if they utilize the data or functionality of another asset. If this asset fails, the operation of the dependent asset will be impacted.
- *Coupling*: Two assets may have both incoming and outgoing dependencies and failure; one of them certainly affects the functionality of the other.

The asset analysis further focuses on the possible cyber dependency level. A cyber dependency of assets is assumed to be a cyber-asset pair (node) interrelation and/or interconnection (edge) aiming to fulfill an electronic service/operation over communication networks. This dependency has evolved due to the purpose of the assets and their relationship with the services. The cyber-asset pair includes a source cyber-asset and a destination cyber-asset [43]. This dependency type can define how a cyber-asset pair is interdependent within the service. Several cyber dependency types are taken into consideration for analyzing the assets: Hosting, Exchange data/information, Storing, Controlling, Processing, Accessing, Installing, Trusted, and Connecting. Each dependency is linked with a dependency access vector, which is capable of defining the dependency within the identified services. To achieve this, the following access vectors are considered: Local (L), Adjacent Network (A), and Network (N). This dependency can change over time, such as from accessing to processing. d-CSRM investigates the possible evolution of these dependencies for risk management.

To demonstrate the cyber dependency, a running example is provided for an organizational context. Assuming there are four cyber assets: A Operating System (OS) $a_{l,m}$, a database of customer records $a_{r,m}$ installed on the previous OS $a_{l,m}$, a web application software $a_{p,t}$ exchanging information with the database $a_{r,m}$, hosted by a web server $a_{q,t}$. The cyber dependency is demonstrated in Table 1.

The outcome of the phase 1 is an asset inventory with a list of properties as shown in Table 2.

**Table 2:** Asset inventory

Asset ID	Asset characteristic	Field	Sub-field
$a_{q,t}$	Asset name		
	Asset product details	Product (CPE URI)	
		Product version	
		Vendor	
		Asset type	Software/software
			OS/information
		Asset sensitivity	Restricted/unrestricted
	User rights	Privileged	Local admin
			Domain admin
		Non-privileged	Local users
			Domain users
	Service level dependency	Type	Business supporting
			Business operation
			Business critical means
			Mission critical means
	Cyber dependency (CD)	Asset dependency level (ADL)	
		Dependency type	Hosting
			Exchange data/information
			Storing
			Controlling
			Processing
			Accessing
			Installing
			Trusted
			Connecting
		Dependency access vector	Local (L)
			Adjacent network (A)
			Network (N)

#### Phase 2: risk assessment

This phase identifies and assesses individual risks related to the assets. The risk assessment context considers the dynamic parameters of the system and security. This phase consists of two steps, which are presented below.

**Step 2.1: Individual threat assessment**

This step identifies and assess the threats that are targeted at services and relevant assets (Table 3). Individual threats can be considered as potential stepping stones to security incidents (deliberate or accidental), which may affect the identified services and assets. The identified threats can be categorized through threat taxonomies and assessed in a qualitative manner using threat scales. Our approach considers the widely known threat catalogue, i.e., Common Attack Pattern Enumeration and Classification (CAPEC) to assess threats relevant to the assets. CAPEC offers distinct set of characteristics such as abstraction, attack likelihood, severity, pattern, IoC, consequence, mitigation, etc., to provide detailed about a threat. In this way, we obtain the list of the threats for each of the previously identified asset that operates in the considered service/system.

The assessment of the threats, assigning a severity level, is based on the approach previously described in [27, 31], which leverages Natural Language Processing (NLP) using a cybersecurity-domain Named Entity Recognition (NER) model. In detail, this method exploits a large Natural Language corpus which includes the history of reported incidents related to those threats, extracted from large collections available online, such as forums, social media, news, and others. A specifically trained NER module based on Secure-BERT [44], a BERT Large Language Model (LLM) pre-trained on a very large CS domain text collection (containing more than 2.2 million documents), preprocessed with a customized tokenizer.

**Table 3:** Threats per asset

Asset	Threats
$a_{k,i}$	$T_1, T_2, T_3,\ldots , T_j$
$a_{k,i+1}$	$T_3, T_4, T_5,\ldots , T_m$
$a_{k,i+2}$	$T_1, T_3, T_4,\ldots , T_n$
$a_{k,i+3}$	$T_5, T_6, T_7,\ldots , T_z$

**Table 4:** Threats assessment scale

Threat scale		Description of threat level
Threat occurrence	Value range (%)	History of incidents (from logs, reports, known catalogues)
Very high	[80–100]	Severe impact on critical services and assets
High	[60–80]	Significant impact on critical services and assets
Medium	[40–60]	Intermediate impact on services and assets and no critical service would be affected
Low	[20–40]	Low impact and no critical service would be affected
Very low	[1–20]	Significant low impact

We fine-tuned the Secure-BERT LLM for the NER task, with the purpose of extracting the mentions of the pairs threat and asset found in each sentence of the natural language corpus. To this end, we created a custom training set, firstly by combining two datasets for NER in the cyber security domain, namely CyNER [45] and APTNER [46], selecting only threat and asset entity types (mapping them with the entities already annotated in these datasets). Moreover, we extended the training set with further documents extracted from the cybersecurity Natural Language corpus, annotated using a semi-supervised approach described in [47].

Using the trained NER NLP-based approach, we are able to identify the possible threats affecting the assets, as reported in the natural language corpora containing history and descriptions of incidents. Then, we can assign a threat-level to each one of them, following the threat scale shown in Table 4. In detail, we defined the threat scale correlating the threat’s level to the percentage of its occurrence in our corpus, assuming that if a threat is more frequently mentioned for an asset, its threat level must be higher. In this way, we defined five different threat levels, from *Very High* to *Very Low*.

In summary, the outcome of the step is the list of detected threats for each asset that operates for the provision of each identified service. Each threat is listed along with a CAPEC ID, a CAPEC category that will be used to rate the threat, and a set of available characteristics that is both informative about the threat and able to procure material for extensions on the methodology throughout the second iteration. Essentially, for each asset $a_{k,i}$ identified in the context of a service all threats $(T_i, a_{k,i})$, a corresponding level is assigned, following the threat scale defined in Table 4.

**Fig. 3:**
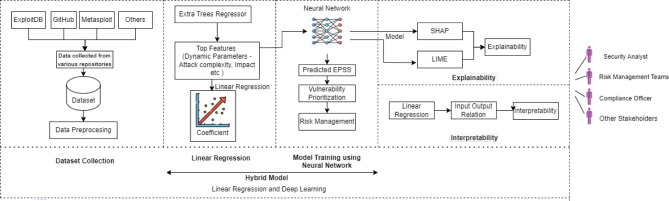
Hybrid model for vulnerability prioritization with explainability and interpretability

**Step 2.2: Individual vulnerabilities prioritization**

The aim of this step includes identifying and analyzing confirmed vulnerabilities concerning the assets that can be leveraged to achieve an attack. For this purpose, we utilize CVEs, with reference to the information that can be sourced from the NVD, including CVE ID, base score, and CVSS vector. The NVD methodology quantifies vulnerabilities using three vectors: Base, Temporal, and Environmental. However, these vectors do not focus on the relative exploitability that can be obtained for specific kinds of assets. In response, the Exploit Prediction Scoring System (EPSS) is employed to select the vulnerabilities in question. EPSS integrates exploit attributes with temporal attributes from sources such as ExploitDB, Rapid7, Metasploit, and GitHub to forecast potential exploitation within one year of the vulnerability disclosure.

d-CSRM aims to adopt the EPSS score from the widely published dataset to prioritize vulnerabilities. However, the EPSS score exhibits certain limitations. Namely, the accuracy of the score substantially depends on data quality, model bias, and the presence of exploitability information. For instance, the integration of information from different sources to compile exploitation-related data can compromise the quality of the data and, consequently, the decisions made by models. Further, the AI model used for the prediction of the EPSS score may present biases. This may lead to a gap between the predicted results and the actual results, potentially causing the EPSS score to be overpredicted or underpredicted.

To mitigate these issues, it is vital to periodically train the AI models used in the prediction of the EPSS score. More training with a good quantity, variety, and up-to-date data is beneficial to fine-tune the model and eliminate biases, enhancing the accuracy of the predictions. The inclusion of feedback mechanisms, where the model’s predictions are checked against actual instances of exploits, can also improve the models. With an emphasis on these variables in training practices, the accuracy and efficiency of the EPSS score in determining the priorities of risk can be significantly enhanced. Additionally, the “black box” problem occurs in AI models, implying that users cannot understand how the models arrive at their decisions. This lack of transparency can make it difficult to trust the model’s predictions. To solve these problems, d-CSRM uses a new hybrid model combining linear regression and deep learning along with Explainable AI (XAI). This approach leverages the strengths of both techniques to achieve better and more accurate EPSS score predictions. While linear regression helps in understanding the linearity between the features, deep learning learns the complex and nonlinear relationships between features. These work hand in hand to increase the accuracy and credibility of the predictions, thereby enhancing the efficacy of the vulnerability management framework. For the purpose of explainability, the SHAP (SHapley Additive exPlanations) framework is utilized, which provides insights into the decision-making process of the model. The following explains each of the steps shown in Fig. 3 in detail in the proposed approach.

**Sub-step 2.2.1: Feature selection**

The step 2.2 can be further divided in sub-steps. The first one is to employ the Extra Trees Regressor to perform feature selection. The Extra Trees Regressor is a type of ensemble learning method that carries out the building of multiple decision trees and then arriving at an average of these. Extra Trees Regressor is similar to the random forest, but it does not consider a random set of features for splitting but splits feature randomly in the entire range which also reduces variance. The execution of this step begins with data preprocessing where data cleaning involves handling of the missing values and normalization of the data. The Extra Trees Regressor is then trained on this dataset to calculate the feature importance. According to these importance scores features are ranked and the features with high importance scores are chosen for further analysis and for building the models, in this way, the features that are less important are filtered out. Additionally, linear regression is used to extract the coefficients of the selected features. This is useful in establishing the correlation between the features and the target variable since it gives a clear insight on how each feature has a bearing on the EPSS score obtained. Applying both Extra Trees Regressor for the feature selection and linear regression for the coefficient analysis helps to make sure that the most important features are used to make the model more efficient and dependable.

**Sub-step 2.2.2: Vulnerability prioritization**

The second sub-step is the prediction of the EPSS score and the prioritization of vulnerabilities. The features chosen from Step 1 are utilized to train the deep learning model, which is designed to estimate the EPSS score, a measure of the likelihood of being exploited. The multiple layers in the deep learning model provide a nonlinear transformation of features, capturing their effect on vulnerability exploitation. After the EPSS scores are predicted, vulnerabilities are prioritized based on these scores. Such prioritization is effective in decreasing the risks that threaten the organization, thereby improving the overall security measures. To increase the relevance of the vulnerabilities, we use a vulnerability scale (1–5) derived from the probability of exploitation (0%-100%) within the next 12 months. For instance, if our hybrid model suggests that a given vulnerability is likely to be exploited by 75%, this would fall under the level defined as ‘High’ on the vulnerability scale. Therefore, it would be addressed based on its criticality, implying that it is highly susceptible to exploitation and poses a high risk. This approach enhances the effectiveness of vulnerability management by leveraging artificial intelligence and data analysis. By prioritizing vulnerabilities according to their EPSS scores, organizations can focus their resources on addressing the most critical threats, thus improving their overall security posture. The vulnerability scale is summarized in Table 5.

**Table 5:** Vulnerability assessment scale

Vulnerability level	Range of probability of exploitation	Description
Very high (5)	$>80\%$	There is greater than 80% probability of successful exploitation of specific vulnerability within the next 12 months
High (4)	60–80%	There is a 60–80% probability of successful exploitation of specific vulnerability within the next 12 months
Medium (3)	40–60%	There is a 40–60% probability of successful exploitation of specific vulnerability within the next 12 months
Low (2)	20–40%	There is a 20–40% probability of successful exploitation of specific vulnerability within the next 12 months
Very low (1)	$<20\%$	There is a less than 20% probability of successful exploitation of specific vulnerability within the next 12 months

This approach also promotes dynamic risk management because the system adjusts its decisions as new information becomes available, and the primary threats evolve. Data is processed in real-time, meaning that the EPSS scores are continuously updated, and the priorities for the identified vulnerabilities change accordingly. The incorporation of this dynamic capability enables organizations to constantly counteract risk factors and address them as they occur, in addition to predicting future risks. This continuous evaluation and application of security measures dramatically increase the organization’s capacity to deal with risks in a rapidly evolving threat environment. By maintaining up-to-date vulnerability assessments and dynamically adjusting priorities, organizations ensure that their security measures are always relevant and effective. This proactive stance on risk management helps in mitigating potential threats before they can cause significant harm, thereby enhancing the overall resilience and security posture of the organization.

The outcome of this step is a vulnerability inventory enlisting the detected vulnerabilities per relevant asset that operates in the context of each identified service. Furthermore, assessments with mean squared error (MSE) and R-squared ($R^2$) coefficients are used to assess the model’s effectiveness. MSE calculates the mean square error of the difference between the actual and predicted EPSS scores with the lower value pointing to higher accuracy. RÂ² estimates the amount of the variance of the EPSS scores in the model, with values closer to 1 implying good accuracy of the model. These provides information on how accurate the model is in predicting and the quality of the data, thereby ensuring its reliability and effectiveness in the vulnerability prioritization.

**Sub-step 2.2.3: Vulnerability prioritization**

This sub-step aims to increase the model’s explainability through XAI techniques primarily involving the SHAP framework. SHAP was adopted for this research because it is an effective technique in explaining individual predictions of AI models. It is based on cooperative game theory and its goal is to fairly divide the prediction of the model among features. SHAP values are derived for each feature and reflect the amount of contribution it offers towards a particular prediction, making them useful in understanding the whole process behind the model’s decision. There are various SHAP explainer algorithms that can be used to calculate the SHAP values for the models. In this research, the Deep Explainer was used. The Deep Explainer is specifically intended for deep learning models for instance like the neural networks. It measures the relevance of each attribute by using the architecture and the gradients of the model. This method provides an understanding of how the deep learning models arrive at decisions, since it provides full information about the feature contributions for specific prediction. SHAP helps to open the ‘black-box’ of AI models such as neural networks and ensemble methods, thus, making sure they can be used as trustworthy components in decision-making. The SHAP value for a feature is calculated as shown in Eq. 1.

1 $$\begin{aligned} \Phi _i = \sum _{S \subseteq N \{i\}} \frac{|S|!(|N|-|S|- 1)}{|N|!}[f(S\cup \{i\})-f(S)] \end{aligned}$$

where,

- $\Phi _i$ is the SHAP value for feature *i*;
- *N* is the set of all features;
- *S* is a subset of*N* that does not contain feature *i*;
- *f*(*S*) is the prediction for the subset *S*;
- |*N*| is the total number of features.

SHAP values are also used to attain both global and local explainability of a model’s behavior. It also allows deriving feature importance not only from the whole dataset, but also from the particular instances of this dataset. SHAP values offer a unified measure of feature importance based on each feature’s contribution to the model’s output.

- ***Global Importance***: global importance provides information on which features are more significant across the entire data set. When the SHAP values are summed over all the instances, it gives a general idea about which of the features are the most important on average. This aids in identifying the general impact of each feature in the model, which gives some clue to the variables dominating the model’s actions.
- ***Local Importance***: Local importance is concerned with providing explanations for each feature for a particular instance prediction. With respect to a particular instance of the dataset, SHAP values offer the distribution of all features to explain how each feature influences the forecast. This gives a detailed look at how a particular prediction was made, thus increasing confidence in the activities of the model.

LIME is an interpretability technique that is meant to explain the predictions of any black-box AI model. It addresses one of the key challenges in AI, the issues related to the explainability of the models. The process of LIME involves several steps that help in the provision of accurate explanations. First, it produces the perturbed samples, which is the set of samples that are almost similar to the instance being explained. These perturbations allow to investigate the region of the decision plane of the complex model near the instance. The complex model then predicts outcomes for each of the perturbed sample giving a set of predictions that describe how the model behaves on the instance. To focus on the region of interest, LIME weights the perturbed samples based on their proximity to the original instance, giving higher weights to more similar samples. This weighted set of samples and their predictions are used to train an interpretable model, such as a linear model or decision tree, that mimics the complex model’s local behavior. The coefficients or decision rules of this interpretable model provide a clear insight into which features and to what extent they contributed to the complex model’s prediction. This localized approach ensures the explanation is both relevant and comprehensible.

LIME creates an optimization problem to balance being accurate to the complex model and easy to understand. Given an instance *x*, perturbed samples $z'$, a complex model *f*, and an interpretable model *g*, the goal is to find an interpretable model that approximates *f* well around *x*.

This is achieved by solving the following optimization problem as shown in Eq. 2:

2 $$\begin{aligned} \xi = arg min_{g \in G} L(f,g,\pi _x) + \Omega (g) \end{aligned}$$

where, $L(f,g,\pi _x)$ is a loss function that quantifies how well the interpretable model *g* approximates the predictions of the complex model *f* for the perturbed samples, weighted by their proximity to $x(\pi _x(z'))$. The term $\Omega (g)$ is a regularization term that ensures the simplicity of the interpretable model, thus enhancing its interpretability.

In practice, the loss function is often specified given in Eq. 3:

3 $$\begin{aligned} L(f,g,\pi _x) = \sum _{z' \in Z} \pi _x(z')(f(z')-g(z'))^2 \end{aligned}$$

This equation is the sum of the squared difference between the outputs of the complex model *f* and the interpretable model *g* over all the samples perturbed from the original data. In this way, LIME reduces this generic loss to guarantee that the interpretable model mimics the black box model’s behavior in the local vicinity of the instance.

**Sub-step 2.2.4: Interpretability using linear regression**

Interpretability is one of the main components of AI, especially as the models become more complex and the task they perform increasingly important. It can also be defined as the degree to which a human being can understand why a certain decision was made at by a model. It is necessary for several reasons, such as building trust, ensuring accountability, and producing practical recommendations. In this regard, Linear regression is one of the most explainable AI models because of the simplicity it possesses and the nature of linear correlation between variables that it assumes [48]. All elements of a linear regression model are easily interpretable and therefore this model is very useful for explanation of data. The model assumes a linear relationship between the inputs and the output, which can be represented by the Eq. 4:

4 $$\begin{aligned} y = \beta _0 + \beta _1 x_1 + \beta _2 x_2 +\cdots + \beta _n x_n \end{aligned}$$

where *y* is the predicted output; $\beta _0$ is the intercept, representing the baseline value of *y* when all input features are zero; $\beta _1, \beta _2,\ldots , \beta _n$ are the coefficients (weights) corresponding to each input feature $x_1, x_2,\ldots , x_n$.

This structure has several beneficial effects on the interpretability of the AI models. First, the significance of each coefficient $\beta _n$ indicates the strength and direction of the relationship between an input feature $x_n$ and the output *y*. An increase in a feature will lead to an increase in the output if it has a positive coefficient; on the other hand, a negative coefficient indicates that the output will decrease if the feature increases [49]. Second, the size of each coefficient depicts the degree of the feature’s influence on the output, with greater absolute values suggesting enhanced significance. Further, it is easier to comprehend the role played by each feature in the final decision meaning the roles of the various features in the determination of an outcome.

**Step 2.3: Risk assessment** The final step of phase 2 assesses both individual and cascading risks based on threat, vulnerability, and impact levels using two sub-steps. d-CSRM considers the recorded CVSS CIA impact attributes for impact assessment by examining the confidentiality, integrity, and availability impact levels. CVSS rates the vulnerability on a three-tier scale: None, Low, and High. Table 6 presents the mapping that follows these three scales and converts them into a unified five-scale range from “Very Low” (VL) to “Very High” (VH). This conversion provides a single estimation for the overall impact of a specific asset/vulnerability combination.

**Table 6:** Impact level calculation

C	None			Low			High		
A	I								
	None	Low	High	None	Low	High	None	Low	High
None	VL	VL	L	L	L	M	M	M	H
Low	VL	L	M	L	M	H	M	H	VH
High	L	M	M	M	H	H	H	VH	VH

**Sub-step 2.3.1 Individual risk assessment** The individual Risk Assessment is calculated by the product of threat, vulnerability and impact level as shown in Eq. 5. The risk calculation mapping based on the Eq. 1 is presented in Table 7.

5 $$\begin{aligned} R_{I L A_{i,j}} = T_L V_L I_L \end{aligned}$$

where $A_{i,j}$ is the *ith* asset for the *jth* service; $R_{I L A_{i,j}}$ is the Individual Risk level for the asset $A_{i,j}$; $T_L$, $V_L$ and $I_L$ are respectively the threat level, the vulnerability level and the impact level for $A_{i,j}$, calculated as explained above.

**Table 7:** Risk calculation mapping

$T_L$	Very low					Low					Medium					High					Very high				
$V_L$	VL	L	M	H	VH	VL	L	M	H	VH	VL	L	M	H	VH	VL	L	M	H	VH	VL	L	M	H	VH
$I_L$	Risk level																								
VL	VL	VL	L	L	L	VL	L	L	L	M	VL	L	L	M	M	L	L	M	M	M	L	L	M	M	M
L	VL	L	L	L	M	L	L	L	M	M	L	L	M	M	M	L	M	M	M	H	L	M	M	H	H
M	L	L	L	M	M	L	L	M	M	M	L	M	M	M	H	M	M	M	H	H	M	M	H	H	H
H	L	L	M	M	M	L	M	M	M	H	M	M	M	H	H	M	M	H	H	H	M	M	H	VH	VH
VH	L	M	M	M	H	M	M	M	H	H	M	M	H	H	H	M	H	H	H	VH	M	H	H	VH	VH

**Sub-step 2.3.2 Cascading risk assessment** This sub-step assesses the cascading risks associated with multiple assets and their related dependencies to generate an attack path. Cascading risks could have a catastrophic impact due to the dependency among multiple assets for a successful attack. Such risks rely heavily on the attack path generated by the execution of vulnerabilities within the dependent assets. Therefore, the attack path initiates with source and target point assets, followed by the vulnerability chain for a successful attack campaign. The vulnerability chain consists of multiple vulnerabilities within the dependent assets and acts as a precondition to materialize a cascading risk. It demonstrates a series of exploitations of vulnerabilities using appropriate access vectors and the escalation of privileges by following the CVSS metrics. In this regard, a threat actor needs to combine the exploitation of multiple vulnerabilities that exist within the attack path of interconnected assets, starting from entry point assets to the target point asset. d-CSRM calculates the cascading risk by multiplying the propagated vulnerability level occurring from source and target point assets and associated threat and impact levels, as shown in Eq. 6.

6 $$\begin{aligned} R_{CL} = T_L PV_L I_L \end{aligned}$$

where $R_{CL}$ is the cascading risk level; $T_L$ is the threat level: $PV_L$ is the propagated vulnerability level for $A_{i,j}$; $I_L$ is the impact level.

#### Phase 3: risk controls

This final phase of d-CSRM develops an effective risk management strategy to mitigate the identified individual and cascading risks. This is a decision-making process to choose the right control strategy and suitable recommended controls. The phase begins with reviewing the vulnerabilities and threats related to the identified individual and cascading risks as an initial baseline for reasoning to choose the right control strategy. It considers risk factors analysis to check whether the identified strategy can eliminate or reduce the individual and cascading risks as well as improve overall cybersecurity. The risk factors are the causes for a risk, which include individual vulnerabilities, vulnerability chains, and threats that impact the assets. The individual risk factor analysis focuses on the vulnerabilities common to most of the assets, and the treatment strategy should prioritize these vulnerabilities to determine the control. The cascading risk factors analysis needs to investigate the vulnerability chain so that the identified control can mitigate the potential cascading spread of vulnerabilities from one asset to another. The strategy should be identified and implemented according to the most prioritized vulnerabilities, significant threats, and risk levels. This phase includes two key steps:

- ***Step 3.1***: Identify common attack points: This step investigates the common attack points by examining the risk factors for both individual and cascading risks, specifically prioritized vulnerabilities, relevant threats, and their frequency of occurrence. A common attack point also identifies the assets that need immediate attention to reduce exploitation and cut off most attack paths. It could be single or multiple attack points depending on the number of vulnerabilities that are exploited within the interdependent assets.
- ***Step 3.2: Identify and prioritize controls***: This final step aims to identify and prioritize the recommended controls that could prevent the exploitation of the vulnerability chain. The controls are categorized based on their types: technical, administrative, physical, and compliance. The controls should focus on the assets related to the common attack points so that they can cut off the attack path. It may not be realistic to tackle all vulnerabilities; therefore, the focus should be on the prioritized ones. Security standards provide a recommended list of controls, which can be used as a reference point to identify the appropriate controls. Some controls require immediate implementation and need to be prioritized for risk mitigation.

## Experiments and results

This section presents the experimental assessment and the results obtained using the proposed hybrid model that utilizes the strength of both linear regression and deep learning. The purpose of the experimental assessment is:

- To attain the most important features using the extra trees regressor.
- To use the features for the further training if the deep learning models to predict the EPSS.
- To prioritize the vulnerabilities from the new predicted EPSS.

### Dataset description

CVEjoin [16] remains as a widely used dataset that has been collected and cleaned up to its finest detail to change the way vulnerability assessments are executed. CVEjoin differs from the traditional ways of vulnerability assessment that is based solely quantitative measures as CVSS and recognizes the need to consider the rest of the threat spectrum and the peculiarities of certain assets. This dataset includes intrinsic vulnerability characteristics derived from NVD, security feeds & social networks depending on the spectrum of the sources. In research that is complex and encompasses a number of objectives, CVEjoin is a most valuable resource for research purposes. CVEjoin dataset and the scripts used for its construction are freely available on GitHub to promote community-driven innovations in the sphere of information security.

**Table 8:** Key features selected using Extra Trees Regressor

Rank	Features	Coefficient
1	user_interaction_REQUIRED	0.006016
2	attack_vector_NETWORK	0.026514
3	integrity_impact_PARTIAL	$-$ 0.032531
4	attack_complexity_LOW	$-$ 0.023895
5	integrity_impact_NONE	$-$ 0.010045
6	availability_impact_NONE	$-$ 0.051403
7	availability_impact_HIGH	$-$ 0.041109
9	attack_complexity_MEDIUM	0.009663
10	confidentiality_impact_NONE	$-$ 0.013366

The dataset includes over 200,000 vulnerabilities, providing extensive coverage for research and analysis. Each vulnerability entry is characterized by 23 attributes, allowing for detailed and nuanced analysis. Some of these attributes are listed below.

- **cve_id**: CVE identifier
- **cwe**: Common Weakness Enumeration identifiers related to vulnerability.
- **cvss_type**: Type of CVSS used.
- **attack_vector**: The context by which vulnerability exploitation is possible.
- **attack_complexity**: Complexity of the attack required to exploit the vulnerability.
- **privileges_required**: Level of privileges an attacker must possess to exploit the vulnerability.
- **user_interaction**: Whether user interaction is required for exploitation.
- **confidentiality_impact**: Impact on confidentiality.
- **integrity_impact**: Impact on integrity.
- **availability_impact**: Impact on availability.
- **base_score**: The base CVSS score representing the intrinsic characteristics of the vulnerability.
- **exploitability_score**: Exploitability sub-score from CVSS.
- **impact_score**: Impact sub-score from CVSS.
- **exploit_count**: Number of known exploits for the vulnerability.
- **attack_type**: Type of attack
- **epss**: Exploit Prediction Scoring System score.

**Table 9:** Summary of the evaluation of the performance of the model

Parameter	Value	Description
Mean squared error (MSE)	0.008498	Indicates the average squared difference between the actual and predicted EPSS values (a lower value often signifies better predictive accuracy)
$R^2$	0.653225	Means that approximately 65% of the variance in EPSS is explained by the model’s features

### Implementation of proposed hybrid model for vulnerability prioritization

#### Feature selection

Table 8 shows the key features that were selected using the Extra Trees Regressor as explained in Sect. 4. The data is then divided into training and testing sets, and then a multiple linear regression model is fit on the training data. Then the output of the regression model is demonstrated in the form of a set of coefficients to explain the significance of each feature with reference to the target variable. For instance, the coefficient for ‘*attack_vector_NETWORK*’ is 0.026514, indicating a positive impact on the ‘*epss*’ value; this means that if the attack vector is ‘NETWORK‘, the ‘*epss*’ value increases by approximately 0.026514 units, reflecting higher exploitability. Conversely, the coefficient for ‘*availability_impact_LOW*’ is $-$0.051953, indicating a negative impact on the ‘*epss*’ value; this means that if the availability impact is ‘LOW’, the ‘*epss*’ value decreases by approximately 0.051953 units, reflecting lower exploitability. These examples demonstrate how different features, such as attack vector and availability impact, influence the ‘*epss*’ value, with positive coefficients indicating a direct relationship and negative coefficients indicating an inverse relationship.

#### Vulnerability prioritization

Once the key features have been chosen, a neural network model is compiled with Adam optimizer and trained on the training dataset. The evaluation of the model is performed with mean squared error (MSE) and R-squared ($R^2$) values. The summary of the evaluation of the performance of the model is given in Table 9.

According to the Table 9, both MSE and R^2^ values confirm that the proposed model offers a certain degree of predictability. Specifically, the low MSE value demonstrates that the predicted EPSS score closely aligns with the actual score, while the higher R^2^ value indicates the ability of the model to explain the variance between the actual and predicated EPSS scores. Notably, a higher R^2^ value depends on several factors, including the quality of input features, model complexity, and hyperparameter tuning. These factors are independent from each other, therefore improving the quality of input feature using diverse and real-time data, does not impact the others. Furthermore, preprocessing data by addressing missing values or encoding categorical variables, and feature scaling all have an impact on R^2^. Model complexity is also essential because a model that is too simple is prone to underfitting, while an overly complex model can overfit and not generalize well. In our case, we explored model fine-tuning as a part of hyperparameter tuning by adjusting parameters such as batch size (increased to 128), activation function (switching from ReLu to Leaky ReLu), dropout rate (increasing from 0.2 to 0.3) and number of epochs (extended to 500) in the neural network. These refinements contributed to better generalization and improved model performance, allowing to increase the R^2^ value from 0.135 (initially obtained without hyperparameters tuning) to 0.653.

Subsequently, to prioritize the vulnerabilities, the predicted EPSS scores are computed on the entire dataset. These vulnerabilities are then ordered in decreasing order according to the predicted scores and the 20 most critical ones are chosen as shown in Table 10 with vulnerability level very high, high and medium. These top vulnerabilities are then combined with the original CVE and CWE as indicated in Table 10 for further output. The three prioritized vulnerabilities on the basis of the calculated EPSS scores are CVE-2020-8515 with predicted EPSS score 0.952958, CVE-2021-26855 with predicted EPSS score 0.900229, and CVE-2018-0776 with predicted EPSS score 0.890287. CVE-2020-8515 relates with DrayTek routers product and prone to multiple command-injection vulnerabilities(CWE-78) due to insufficient validation of user-supplied input. CVE-2021-26855 relates with Microsoft Exchange Servers due to server-side request forgery (CWE-918) which allows attacker to send arbitrary HTTP requests. CVE-2018-0776 relates to Microsoft Edge product due to scripting engine vulnerability which allows remote code execution(CWE-787).

The dynamic components of the models are:

- Neural network model training.
- Calculation of EPSS scores for the full dataset.
- Dynamic ranking of vulnerabilities based on EPSS scores.
- Adaptive model training with new data.
- Prioritization of threats according to current EPSS scores.
- Dynamic incorporation of prioritized vulnerabilities with CVE and CWE databases.

All these dynamic elements can potentially be used for risk management. Applying dynamic indicators during the assessment of the model’s efficiency provides organizations with a means of scrutinizing the tool’s effectiveness and reliability in detecting the exploitation of vulnerabilities regularly. This approach of prioritizing the threats according to their current EPSS scores will help organizations to follow the learning hypothesis, thereby improving their security standing. This allows organizations to always assess their risk and vulnerabilities and defend against new risks as they emerge. Thus, incorporating these activity change characteristics enables the enhancement of real-time and reliable threat risk management that is flexible to the ever-changing threats.

**Table 10:** Vulnerability prioritization from hybrid model

CVE	CWE	Predicted EPSS	Vulnerability level
CVE-2020-8515	[CWE-78]	0.952958	VH
CVE-2021-26855	[CWE-918]	0.900229	VH
CVE-2018-0776	[CWE-787]	0.890287	VH
CVE-2018-4878	[CWE-416]	0.877147	VH
CVE-2018-0953	[CWE-787]	0.823484	VH
CVE-2020-5902	[CWE-22]	0.815564	VH
CVE-2020-8644	[CWE-94]	0.794113	H
CVE-2018-17456	[CWE-88]	0.784725	H
CVE-2020-10220	[CWE-89]	0.736111	H
CVE-2021-44529	[CWE-94]	0.728556	H
CVE-2020-10199	[CWE-917]	0.715313	H
CVE-2020-2038	[CWE-78]	0.713794	H
CVE-2019-18935	[CWE-502]	0.666516	M
CVE-2020-8983	[CWE-22’]	0.663765	M
CVE-2019-8385	[CWE-22]	0.641083	M
CVE-2018-19864	[CWE-20, CWE-119]	0.602549	M
CVE-2013-0803	[CWE-434]	0.597067	M
CVE-2018-1000049	[CWE-20]	0.592000	M
CVE-2021-27964	[CWE-434]	0.562293	M
CVE-2018-0986	[CWE-787]	0.559484	M

### Assurance of explainability

#### LIME analysis

**Fig. 4:**
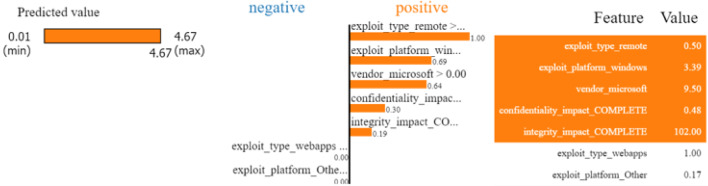
Importance of features using LIME

Figure 4 illustrates the implementation of the LIME method to interpret the prediction of the EPSS value. The LIME method offers a way to understand how the features contributed to the prediction made by the model. The LIME-predicted EPSS value is about 4.67, indicating that the probability or severity of the exploit is high. The LIME explanation describes the importance of different features for this prediction.

**Positive contribution**: The highest positive contribution is the ‘*exploit_type_remote*’ (0.50), which clearly shows that the severity level of a remote code execution is high. The second-highest positive contribution is ‘*exploit_platform_windows*’ (3.39), and sequentially, ‘*vendor_microsoft*’ (9.50) also plays an important role in the indicators’ increase and stresses the role of vulnerabilities that threaten popular platforms and well-known vendors, such as Microsoft. To complement these observations, the features ‘*confidentiality_impact_COMPLETE*’ (0.48) and ‘*integrity_impact_COMPLETE*’ (102.00) also indicate that the model effectively captures the key factors influencing the overall risk level associated with vulnerabilities.

**Negative contribution**: Features such as ‘*exploit_type_ webapps*’ (1.00) and ‘*exploit_platform_Other*’ (0.17) contribute negatively, although their impacts are relatively minor in this case.

The experimental results from LIME not only support the key findings of the model, but also demonstrate the practicality of these findings. Hence, features that contribute positively to the final model prediction allow us to determine which vulnerabilities are more probable to exploit than others, so that risks related to these vulnerabilities can be prioritized using this dynamic risk assessment method. Similarly, features contribute negatively to the EPSS score prediction, allowing for a better understanding of less exploitable vulnerabilities and helping organizations allocate their security resources more efficiently. Furthermore, the LIME model identifies critical impacts, such as impact on confidentiality and integrity breaches, underscoring the urgency for immediate remediation for risk mitigation. These insights can be directly integrated into dynamic risk management workflows by enabling risk-based prioritization of vulnerabilities, guiding patching schedules, informing access control decisions, and enhancing security dashboards for analysts. By embedding such interpretability into AI systems, organizations can respond more intelligently and efficiently to evolving threats.

#### SHAP analysis

**Fig. 5:**
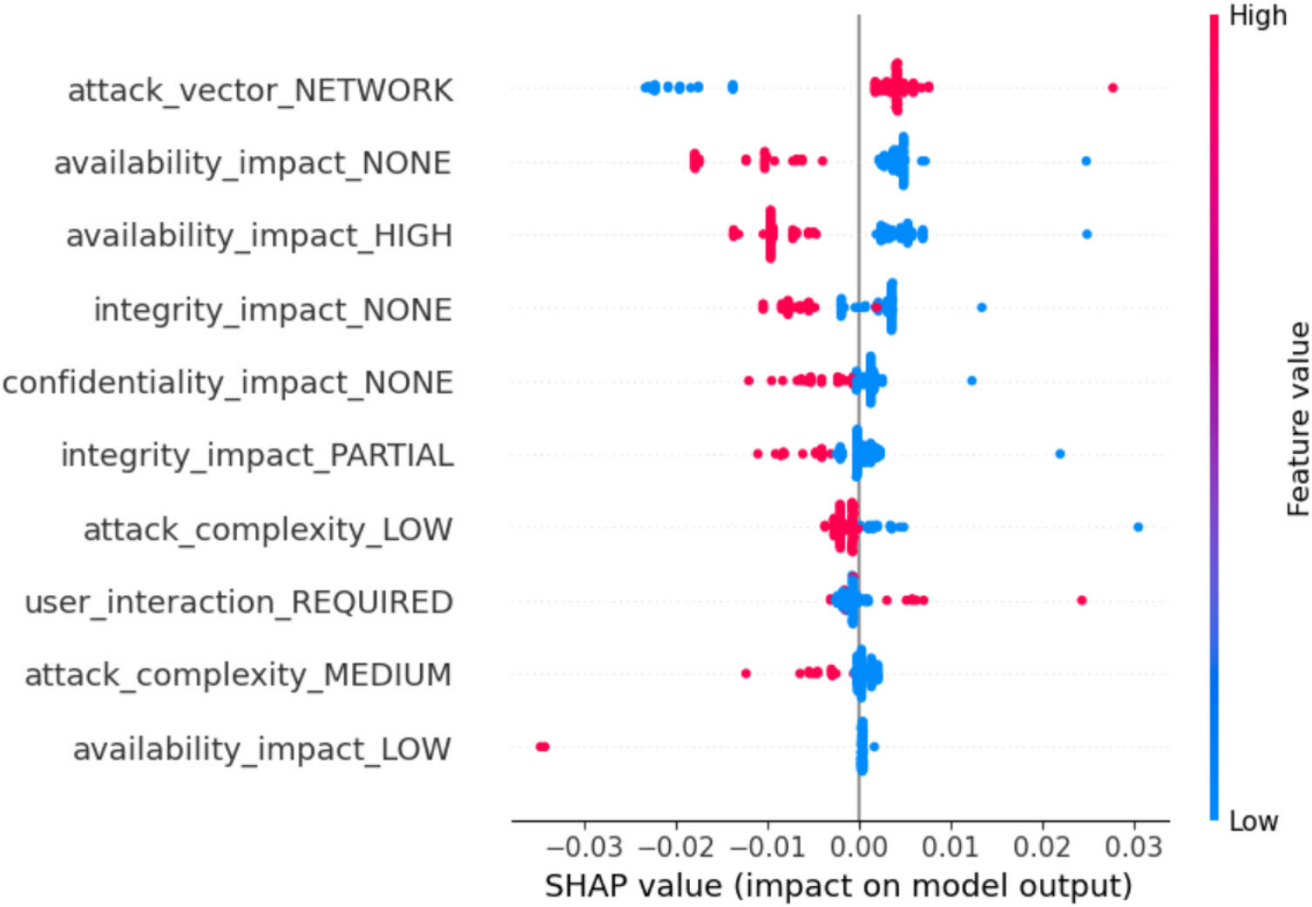
Local importance of features

**Local importance**: Figure 5 shows local feature importance of the model, explaining which features have the highest impact on the decision. The color gradient scale which includes blue and red, are used to illustrate the feature values where blue means low values while red means high values. The ‘*attack_vector_NETWORK*’ stands out as the most influential one of them, as any increase in its value leads to a higher EPSS score. The levels of availability impact (NONE, HIGH) are clearly and significantly different, proving the fact that these impacts are critical to the score. Attack features such as attack complexity, which can either be LOW or MEDIUM and user interaction, which is REQUIRED are critical factors that define the model.

**Fig. 6:**
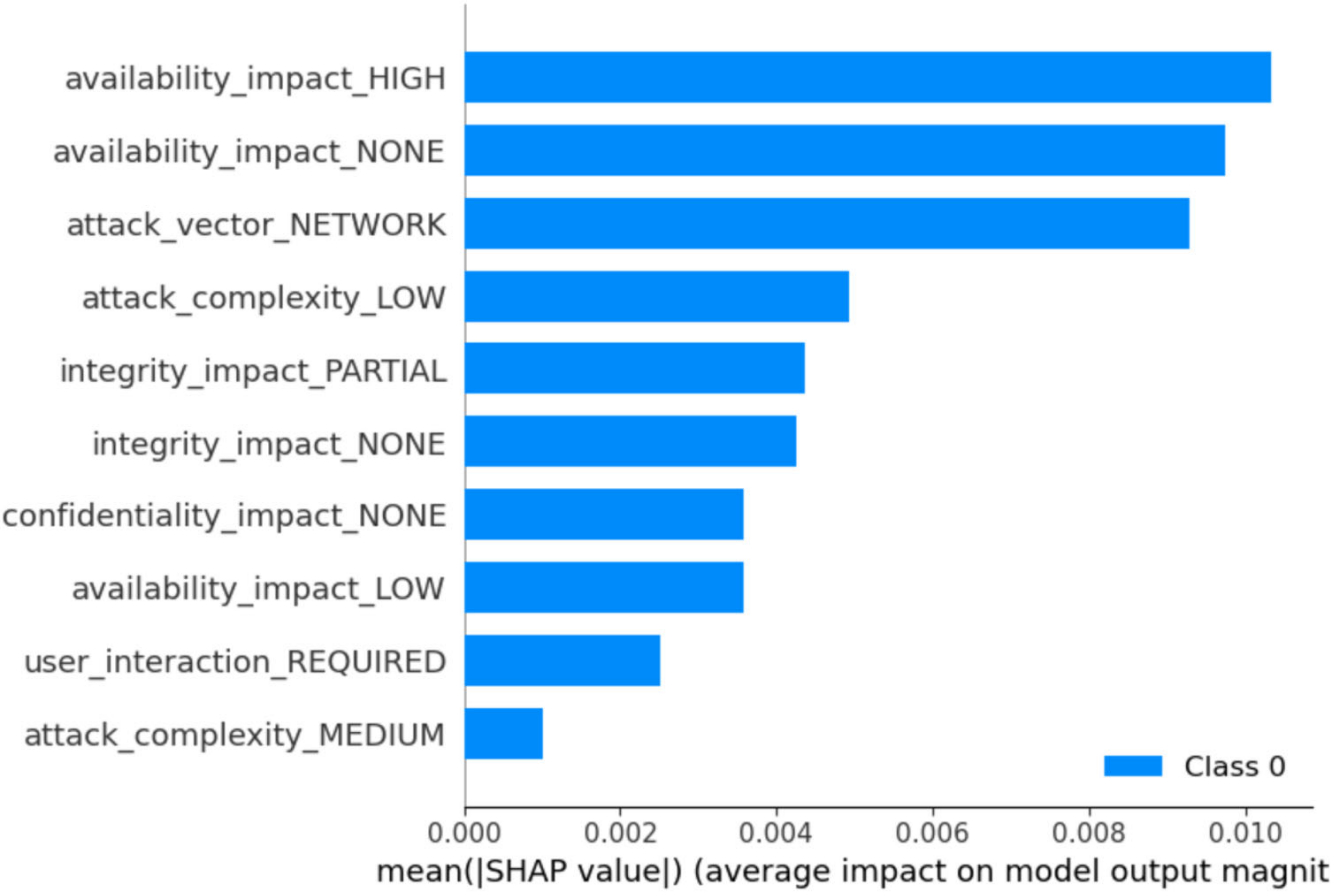
Global importance of features

**Global importance**: Figure 6 illustrates the global importance of features in determining the predicted outcomes of the model, as measured by the SHAP values. The most important feature, ‘*availability_impact_HIGH*,’ suggests that vulnerabilities affecting availability are crucial in shaping the model’s outcomes. Similarly, both ‘*availability_impact_NONE*’ and ‘*attack_vector_NETWORK*’ have significant effects, demonstrating that the lack of availability impact and the presence of network-based attack vectors are important factors in determining the model’s result. Other relevant but less significant attributes are ‘*availability_impact_LOW*’, ‘*user_interaction_REQUIRED’*, and ‘*attack_complexity_MEDIUM*’.

**SHAP summary**: By following the experiment, we have summarized the capabilities of SHAP to support the d-CSRM:

- Both local and global plots highlight the significance of availability impact features, particularly ‘*availability_impact_HIGH*’ and ‘*availability_impact_NONE*’, as the most influential contributors to the prediction of the model. This indicates that the model concentrates on minimizing system downtime and service disruption – most critical in dynamic risk management – and enabling organizations to rank vulnerabilities that can produce costly availability issues for timely and effective mitigation.
- Attack complexity and user interaction are also foremost drivers of the model’s output; the very high positive SHAP value of ‘*attack_complexity_LOW*’ indicates that attacks with low complexity are more probable to be attacked and hence have higher EPSS scores, which facilitate real-time prioritization of such vulnerabilities in dynamic security context.
- The SHAP output gives a clearer view of the contribution of features, showing how different feature values affect the EPSS score and allowing context-based risk assessment and vulnerability patch update for adaptive vulnerability prioritization according to exploitability, exposure, and impact on operation.

### Assurance of interpretability

As discussed earlier in Table 8 in Sect. 5.2.1, the interpretability of linear regression is well-demonstrated by the provided coefficients. Each feature’s coefficient offers a clear and direct understanding of its effect on the predicted outcome: positive coefficients indicate features that increase the outcome, while negative coefficients indicate those that decrease it. The magnitude of each coefficient indicates the significance of the feature, enabling comparison and understanding of which features are most important for affecting the outcome. One of the strengths of linear regression is that it offers a clear and direct correlation between the features and their corresponding outcomes, which is beneficial in terms of interpretation and decision-making. For instance, from the table we can derive that:

- *user_interaction_REQUIRED* (0.006016): this feature has a positive coefficient, meaning that the requirement of user interaction slightly enhances the predicted result. However, since it is small in value, it means that the impact is negligible or almost insignificant.
- *integrity_impact_PARTIAL* ($-$ 0.032531): this feature has a negative coefficient meaning that a partial impact on integrity reduces the expected result. This feature has moderate influence or moderate effect, as denoted by the magnitude of the coefficient value.

Linear regression increases the interpretability of a model as it gives direct relations of features and outcomes and quantifies the impact of each feature while being clear and simple. This is crucial in instances where the users have to convey the model’s outcomes to other users or stakeholders meanwhile making the explanations simple and easy to comprehend. This in turn can lead to effective decision-making and stakeholder engagement.

## Discussion

The organizational digital infrastructure is constantly changing to meet overall business needs, and at the same time, so is the security posture within this infrastructure. Therefore, an effective cybersecurity risk management practice is essential for managing the risks to ensure security and resilience. However, this task becomes more challenging due to the evolving context. Accurate risk assessment requires considering the dynamic parameters from the infrastructure and security context, which is challenging. The proposed d-CSRM contributes to tackle this challenge by considering dynamic parameters for risk management activities. Additionally, the work makes use of AI models to analyze the security related data which can identify and quantify the risk. Additionally, it also incorporates the explainability and interpretability frameworks to make the AI models understandable to all user groups.

### Dynamic parameters utilized for cybersecurity risk management

As stated before, d-CSRM considers several parameters from the digital infrastructure and security context that are evolving over time and are necessary to consider for the risk assessment. These parameters include assets and their dependencies, vulnerability exploitation, impact level, and common attack points.

This allows organizations to gain a deeper understanding of the risk values due to the constant changes in the digital infrastructure and security landscape. Security teams can prioritize their efforts more effectively by making informed decisions, focusing on critical assets, and using insights from explainability and interpretability to justify their strategies. The dynamic assessment of these parameters ensures that the organization’s risk posture is always up to date, enabling proactive detection and response to potential threats. By understanding the interdependencies between assets and analyzing vulnerability exploitation and common attack points, d-CSRM enables organizations to re-evaluate the risks and make quick decisions for risk treatment strategies.

### AI-enabled hybrid models for risk management

The proposed d-CSRM incorporates AI-enabled models for the risk identification and assessment. The combination of linear regression and deep learning models enhances the reliability and interpretability of risk assessments, providing a more effective framework for vulnerability management. Linear regression gives a straightforward view of the associations between features [48], while deep learning can learn the non-linear patterns that are not easily modeled by the traditional techniques [49].

This combination guarantees that the EPSS scores are predicted with high accuracy and the most important vulnerabilities are prioritized. This integration enables the re-estimation of risk values based on critical dynamic evidence, such as vulnerability levels, for an effective real-time risk management practice. From the experiment, one of the top vulnerabilities chosen for prioritization are CVE-2020-8515 and CVE-2021-26855. CVE-2020-8515 is an OS command injection vulnerability that can enable the attackers to run unauthorized commands on the operating system of the host through a vulnerable application. This type of vulnerability, with an EPSS score of 0.952958, is classified as very dangerous because, if executed, it leads to a system compromise. CVE-2021-26855 is a server-side request forgery (SSRF) vulnerability that can make the server send crafted requests to other systems based on attackers’ control. This vulnerability has an EPSS score of 0.900229 and is highly critical because internal systems may be accessed by unauthorized persons, leading to the compromise of crucial information or facilitating other attacks. Thus, maintaining the constant revision of the security metrics and the recalculation of risks helps organizations to remain in the state of alert.

Within the evolving organizational and security context, security risk can rise form any part of the organization at any time. The critical business processes and services are now heavily relying on the digital infrastructure; therefore, risks need to be proactively managed to ensure resilience delivery of these services without any interruption. The proposed AI-enabled hybrid model for the d-CSRM needs to be adopted as a continuous ongoing process into the organizational workflows. It provides the capabilities to understand the organization’s security posture and resilience. Moreover, zero-day vulnerabilities or exploitation of published vulnerabilities can happen any time which may affect the operation and considering d-CSRM as a continuous process can support tackling this evolving security landscape.

### Adoption of explainability and interpretability

Integrating explainability and interpretability into AI-enabled security solution is crucial for trustworthy AI. Although AI models have a strong ability to predict, their black-box nature makes it difficult for users and other related parties to understand the models’ decision-making processes. Explainability and interpretability mitigates this challenge by explaining the decision-making of AI models, especially deep learning approaches. These techniques focus on explaining a model’s outputs in a way that users can understand, thereby fostering trust in AI-based decisions. Furthermore, regulatory compliance is enhanced as d-CSRM along with explainability and interpretability provide clear documentation and justification for risk management decisions. For instance, the experiment revealed that the features ‘*user_interaction_REQUIRED*’, ‘*attack_vector_NETWORK*’, and ‘*integrity_impact_PARTIAL*’ are critical for decision-making. By laying out the process that leads to predictions, explainability and interpretability facilitate stakeholders’ comprehension of why certain vulnerabilities are prioritized. This transparency is essential in cybersecurity, where trust in the system’s outputs is paramount for effective risk management. For example, when explaining the model’s predictions of EPSS scores, explainability can reveal why some vulnerabilities are considered more prone to exploitation than others, based on the features of the model examined.

In this regard, SHAP, LIME, and linear regression have been used to improve the explainability and interpretability of the AI model. When describing the model’s predictions of EPSS scores, SHAP can explain how each feature contributes to the predicted score of a specific vulnerability, while LIME can explain why a certain vulnerability is deemed more severe by the local model approximation. This ensures that the information processing by the hybrid model is feasible and credible, underpinning a sound and dependable approach to vulnerability and risk management.

### Comparison with existing cybersecurity risk management standards and frameworks

We compare the d-CSRM with the existing risk management standards and frameworks, especially works that focus on risk management frameworks and adoption of AI for risk management. The ISO 31000 mainly provides the foundations for the risk management practice using process, framework and principals independent of any sector [20], whereas ISO 27005 also focusses on identifying and assessing risk mainly for information security context [21]. These standards do not provide any detailed guideline and related techniques how the risk should be assessed; moreover, the evolution of security context within an organization are not considered. The integrated cybersecurity risk management framework adopts common security knowledge for the threats and vulnerabilities identification and introduces conceptual language and process for the risk assessment and management [9]. This integrated approach also uses an AI model for the risk type prediction, but in a static manner as the model does not contribute to assess specific risk types. A review of risk assessment methods for the SCADA systems shows that mathematical formula, graphical model-based, and approaches with both qualitative and quantitative calculation are mainly considered by the existing practices [10]. Moreover, these works follow the ISO 31000 for developing the risk management process with impact assessment based on vulnerabilities and threats.

In the context of the adoption of AI for cyber risk management, several works consider different AI models for this purpose. For instance, Natural Language Processing (NLP) is used to obtain threat information from security-related unstructured text available on the Internet [31]. The identified threats are mapped with specific healthcare asset and assessed to prioritize the threat level. However, the threat assessment is not linked with the potential risks; therefore, threat mitigation may not ensure controlling the security risks. BERT based LLM model is used to detect vulnerability from the source code-based data set with operationalization of transparency obligation practice using XAI so that model decision and inner working are well explained. The proposed d-CSRM partially maps with some of these works specifically consideration of ISO standards for the risk management process, adoption of AI model with explainability and interpretability practice. However, d-CSRM is unique compared to these works due to the consideration of dynamic security context which allows to assess the security risk based on the evolving parameters. Moreover, d-CSRM assesses both individual and cascading risk, which certainly enhance the security capabilities of the organization as this allows to understand risk not from individual asset but also from dependent assets. Finally, d-CSPM operationalizes the explainability and interpretability of the hybrid model, which justifies how the model prioritizes specific vulnerability for risk assessment.

## Conclusion

The accuracy of the risk assessment and effectiveness of the overall risk management practice require consideration of the dynamic parameters from the system and security context. Otherwise, uncertainty could increase over time with severe consequences. Hence, dynamic cybersecurity risk management enables organizations to respond appropriately and make quick decisions to mitigate risk. The proposed d-CSRM improves an organization’s overall cybersecurity posture by providing insights into assets and their dependencies, prioritized vulnerabilities, common attack points, and more. d-CSRM allows the reassessment of risk value based on changes in the dynamic parameters using a systemic process with distinct phases. d-CSRM also adopts a hybrid AI model, a combination of linear regression and deep learning models, to support understanding vulnerability exploitation for vulnerability prioritization. This hybrid model leverages the strengths of both methods: linear regression offers interpretability and information about linear interactions between features, while deep learning models capture nonlinear interactions. These two approaches are incorporated into the model to provide more accurate and easier-to-interpret EPSS scores, thereby offering a more effective framework for vulnerability management. This way of prioritization aids in centering the efforts of an organization on the most substantial threats, leading to an improvement in security status.

Furthermore, there is a high need for the explainability and interpretability of the AI models to allow stakeholders to trust and rely on the output of the artificial system. D-CSRM acknowledges this need and utilizes the SHAP, LIME, and linear regression to illustrate the feature importance and why specific vulnerabilities are prioritized. This way of prioritization helps in directing the action of an organization to the really important threats that contribute positively to the improvement of the security status of an organization. This level of transparency allows for the reliable usage of the model’s predictions for prioritizing vulnerabilities and their risk. As a future direction of this work, we intend to consider different datasets with diverse AI models like gradient boosting machines, support vector machines, etc. for EPSS prediction to generalize our findings. The inclusion of transparency obligation will further enhance the trust in the model and promote ethical behaviour. Moreover, dynamic parameters also need to be extended to other areas, such as source code analysis, results of penetration testing, and more, which we intend to consider for comprehensive risk management. We are also planning to adopt a case study from a specific sector to determine the applicability of the framework. This case study will help confirm the applicability of the d-CSRM framework in real-world settings and provide further insights into its practical implementation.

## Data Availability

No datasets were generated or analysed during the current study.
